# Editorial Comment: Overall survival prediction in metastatic castration-resistant prostate cancer treated with radium-223

**DOI:** 10.1590/S1677-5538.0343.1

**Published:** 2020-05-20

**Authors:** Rodolfo Borges dos Reis, Valdair Muglia, Eliney F. Faria

**Affiliations:** 1 Departamento de Cirurgia e Anatomia Faculdade de Medicina de Ribeirão Preto USP Ribeirão PretoSP Brasil Departamento de Cirurgia e Anatomia, Faculdade de Medicina de Ribeirão Preto – USP, Ribeirão Preto, SP, Brasil;; 2 Departamento de Imagens Médicas, Radioterapia e Onco-hematologia Faculdade de Medicina de Ribeirão Preto USP Ribeirão PretoSP Brasil Departamento de Imagens Médicas, Radioterapia e Onco-hematologia. Faculdade de Medicina de Ribeirão Preto – USP, Ribeirão Preto, SP, Brasil;; 3 Hospital Felicio Rocho Belo HorizonteMG Brasil Hospital Felicio Rocho, Belo Horizonte, MG; Brasil; 4 Departamento de Urologia Hospital de Amor de Barretos BarretosSP Brasil Departamento de Urologia, Hospital de Amor de Barretos, Barretos, SP, Brasil

## COMMENT

Bone offers has a favorable environment that stimulates prostate cancer tumor growth in a vicious cycle fed by growth factors released by own osteoblasts (1).

Radium223 (Ra-223) is a radioisotope, it delivers energy radiation to prostate cancer bone metastasis leading to DNA damage. Ra-223 is the only commercially released alpha-emitter that targets osteoblastic bone metastases used for treatment of metastatic castration resistant prostate cancer (mCRPC).

The ALSYMPCA2 Phase III trial (2) compared Ra-223 efficacy versus placebo in 921 patients with mCRPC and symptomatic bone metastases. The study excluded patients with visceral metastases. Were included patients with disease progression (after or during) Docetaxel treatment or unfit to receive chemotherapy. The authors reported clear overall survival (OS) benefit in the Ra-223 arm compared to the placebo arm (14.9 months vs 11.3 months, HR =0.7 [95% CI 0.58–0.83]; P<0.001).

The concept of using Ra-223 earlier in the disease course, in asymptomatic or minimally symptomatic patients, is attractive. It would allow patients to complete all the six cycles of treatment and optimize sequencing with other life-prolonging therapies (3).

The combination of Ra-223, in the earlier stages of the disease, to a second-generation androgen blocker such as abiraterone (ERAS trial), and enzalutamide (PEACE-3 trial) has been reported. The ERAS trial revealed an increased risk of fractures in the Ra-223 arm (9%) versus the placebo group (3%); due to adverse effects and fractures, this combination was discouraged (4). The interim results of the PEACE-3 trial, presented at ASCO 2019, suggested that adding a bone protector (zoledronic acid or denosumab) could reduce the number of fractures, but the final results are still pending.

Until now, in the earlier stage of the disease, no sequencing using Ra-223, alone or in combination, has demonstrated survival benefit. The APCCC 2019 panel recommended the use of Ra-223 sometime during the treatment course in patients with symptomatic mCRPC and bone-predominant metastases with no visceral or bulky lymph node metastases” (panel consensus). To reduce bone fractures, the bone-protection therapy should be started before the use of Ra-223 (panel consensus) (5).

The published literature is vast, and there is no generally accepted method to identify patients with mCRPC who would benefit from Ra-223. Although PSA (Prostate-Specific Antigen), ALP (Alkaline Phosphatase) and lactate dehydrogenase (LDH) are established prognostic biomarkers in Mcrpc (6), they are not predictive of response to Ra-223.

In this series of cases, the authors pointed out that low hemoglobin (Hb) levels, bone marrow involvement and an altered performance status were the main factors related to decreased survival, identifying patients who would benefit from Ra-223 therapy (7).

Whether patients with normal Hb, no bone narrow involvement and with good performance status respond better to therapy or just live longer because of the lower tumor burden (lead-time bias phenomenon) is an open debate. Furthermore, no information about subsequent therapies was provided, making it difficult to drive conclusions regarding OS, the main endpoint of the study.

Despite this limitation, the authors reported a prospective case series in a Latin American population. They were able to demonstrate the safety and effectiveness of this therapy in a short time. Unfortunately, until now, despite the interesting mechanism of action, the Ra-223 position in the treatment sequence is still to be defined.
